# Defending behaviors, bullying roles, and their associations with mental health in junior high school students: a population-based study

**DOI:** 10.1186/s12889-016-3721-6

**Published:** 2016-10-10

**Authors:** Wen-Chi Wu, Shyuemeng Luu, Dih-Ling Luh

**Affiliations:** 1 Department of Health Healing and Health Marketing, School of Healthcare Management, Kainan University, No.1 Kainan Road, Luzhu Shiang, Taoyuan, 338 Taiwan; 2 School of Health Administration, Dalhousie University, 5161 George St. Suite 700, Halifax, NS B3J 1 M7 Canada; 3 Department of Public Health, Chung Shang Medical University, No.110, Sec.1, Jianguo N.Rd., Taichung, 402 Taiwan; 4 Department of Family and Community Medicine, Chung Shan Medical University Hospital, No.110, Sec.1, Jianguo N.Rd., Taichung, 402 Taiwan

**Keywords:** Defending behaviors, Depressive symptoms, Social anxiety, Bullying roles

## Abstract

**Background:**

Students should be encouraged to help prevent or stop bullying. However, defending victims of bullying can impact on mental health. It is not only bystanders who may defend victims, but bullies, victims and bully-victims can also have defending behaviors. Nevertheless, most studies of defending behaviors have been limited to an examination of the reactions of bystanders or those not involved in bullying and have ignored the other players. The aim of this study is to investigate the associations between defending behaviors and mental health among bullies, victims, bully-victims and bystanders.

**Methods:**

Associations among defending behaviors, mental health (including depressive symptoms and social anxiety), and bullying experiences were cross-sectionally examined in 3441 students (13–15 years old.) from 20 randomly selected junior high schools in Taiwan using a self-report questionnaire. SAS 9.3 Survey Analysis procedures were used to conduct descriptive analysis and multiple regression models.

**Results:**

Defending behaviors were associated with bullying roles and were higher in victims than in bullies or bystanders. Defending behaviors were positively associated with social anxiety and depressive symptoms. After stratifying by bullying roles, defending behaviors were positively associated with social anxiety in bystanders, and were positively associated with depressive symptoms in victims and bystanders. However, defending behaviors were not significantly associated with mental health indicators in bullies.

**Conclusions:**

The associations between defending behaviors and mental health varied according to bullying roles. The results suggest that bystanders and victims experience more mental health effects than bullies. Intervention programs aimed at preventing bullying should focus on strategies that minimize social anxiety and depression in victims and bystanders, and urge students to help vulnerable peers during bullying events.

## Background

Bullying is a behavior that involves hurting others through a superior/imbalanced power status [1]. Bullying is common in teenagers. Currie and her colleagues in a cross-national study found a rate of bullying of 9–13 % among 11–15 year olds [2]. In Taiwan, Wu et al. found a rate of bullying of 7 % in 13–15 year olds [3]. Bullying is an important issue as previous studies show that bullying experiences in adolescence can cause long-term health effects [4, 5]. Therefore, early intervention to prevent bullying is vital for adolescent and public health.

Involvement in bullying can be classified as either direct or indirect [1, 6]. Direct involvement is further categorized as being a bully, victim, or bully-victim. There are three types of indirect involvement: being a reinforcer or assistant bully, outsider, or victim defender [7, 8]. The former two encourage bullying and the latter helps to stop it [9]. The present study is focused on defending behaviors that can prevent incidents of bullying.

### Defending behaviors and mental health

When peers intervene against bullying, 57 % of bullying episodes cease within 10 seconds [10]. Therefore, encouraging such peer intervention is an important approach to reducing bullying. However, despite the effectiveness of peer intervention, Hawkins et al. found that although bystanders were present in 88 % of bullying episodes, they only intervened and defended victims in 19 % of cases [10].

Defending behaviors may lead to negative mental health outcomes through the effect of peer pressure or through the experience of being a bystander at a bullying event. Peers who intervene against bullying face enormous peer pressure when helping victims, as well as the concern that they themselves will risk becoming the next target. These concerns can make peers unwiling to intervene in bullying episodes [7, 11, 12]. Some bystanders may even choose to join in the bullying to prevent themselves from becoming a victim [7]. It takes tremendous courage to intervene against bullying. As peer pressure is known to be associated with negative mental health outcomes such as depression [13, 14] and social anxiety [15], it is therefore possible that defending behaviors would also be associated with such mental health outcomes. However, to date no study has explored the relationship between defending behaviors and mental health.

As well as the impact of peer pressure, those who have observed a bullying episode are more likely to have mental health problems (including depression and anxiety) than those who have not observed bullying [16, 17]. Therefore, both the impact of peer pressure and the experience of having observed bullying may put those intervening against bullying at risk of poor mental health. As a result, we aimed to examine the relationship between defending behaviors and mental health (measured by depressive symptoms and social anxiety) in the present study.

### Defending behaviors and bullying experience

It is not only bystanders that may display defending behavior but also bullies and victims. Rivers et al. found that 32.6 % of students reported being a bystander in a bullying episode [16]. However, some bullies (6.7 %), victims (15.2 %), and bully-victims (10.7 %) also reported being bystanders some of the time. Despite this mixing of bullying roles, most studies have confined their examination of bystander reactions to bystanders and have ignored the possibility that all three bullying roles may have bystander reactions.

One group of researchers created an undercover anti-bullying team from bullies and bystanders, and charged this team with the mission of protecting and defending victims [18, 19]. They found that bullies in the team worked with bystanders to help victims and together developed a kind of empathy for others, concern for victims and an atmosphere of being open to differences [19]. This demonstrates that bullies can show defending behavior under certain conditions, just like those with other bullying roles. However, past research has focused only on the defending behaviors of bystanders and has rarely examined such behaviors in those with other bullying roles.

### Defending behaviors and mental health by bullying role

It is possible that the relationship between defending behaviors and mental health may vary according to the role played in the bullying experience. The psychological pressure faced by bullies displaying defending behaviors may be less than that of bystanders and victims. Different participant roles in bullying may also be associated with different social status, communication skills, and degree of empathy. For example, a bully’s social status may be superior to that of a victim [20, 21]. The social skills of victims may also be worse than that of bullies and bystanders [22]. Bullies may have a higher degree of self-centeredness than other players [23]. Social status, communication skills and empathy are all related to intervening against bullying [7, 21, 24]. Therefore, due to variation in these characteristics by bullying role, the frequency of defending behaviors and related mental health outcomes could also be different. To date no study has explored the relationship between defending behaviors and mental health and how this differs by bullying role.

Different roles in a bullying episode can be associated with different mental health status [5, 25], including greater social anxiety and depression in victims compared to other bullying roles [4, 26, 27]. Therefore, in order to prevent interference from different bullying roles, we stratified the analysis of relationships between defending behaviors and mental health by bullying roles.

### Hypotheses

Based on the above literature, the present study has the following hypotheses: Hypothesis 1: Defending behaviors will vary according to bullying roles (bully, victim, bully-victim or bystander). Hypothesis 2: Defending behaviors will show a significant association with mental health. Hypothesis 3: The association between defending behaviors and mental health will vary according to bullying roles.

To our knowledge, this study is the first to examine the relationships among defending behaviors, mental health and bullying roles. Our results will be of use in the prevention of schoolyard bullying. For example, if our findings confirm an association between defending behaviors and poorer mental health, future efforts to encourage peer interventions in bulling should work hard at creating a friendly environment to reduce the negative mental health effects associated with defending behaviors. In addition, if our findings confirm that the mental health problems of bullies are lower in those with defending behaviors, when developing interventions an important goal would be how to make bullies themselves willing to intervene against bullying.

## Methods

### Study design and participants

This is a cross-sectional study of 7^th^ to 9^fh^ graders (approximately 13–15 years old) from public junior high schools in Taiwan, 2010. A multistage, random cluster sampling procedure was used. First, five schools were systematically sampled from one of the four geographical areas in Taiwan (North, Central, South, and East). Second, within each of the 20 selected schools, two classes from each grade were randomly selected. A total of 3721 students from 120 classes were invited to participate. Of these, 3441 students completed a self-report questionnaire in their classroom supervised by 22 trained college students, giving a completion rate of 92.50 %.

As those experiencing bystander reactions must have been a bystander in a bullying episode, we excluded all participants who reported that they had not witnessed any of the 13 types of bullying episodes in the bystander experience scale. We excluded 453 students (13.23 %) who reported that they had never observed or noticed any kind of bullying and 116 participants with incomplete data. The final analytic sample consists of 2872 students. There were no statistically significant differences between participants with complete (*n* = 2872) or incomplete (*n* = 116) data with regards to bullying experiences, defending behaviors, social anxiety score, depressive symptoms score and other related variables. There was, however, a significant gender difference as boys were more likely to have incomplete questionnaires than girls.

### Measures

The questionnaire contained three sections: personal information, family background and bulling experiences. The section on bullying experiences included perpetration, victimization, and observation, and was developed based on the literature [1, 6, 8, 28]. The instrument was modified according to the results of content analysis of 10 focus group discussion sessions (5 groups of 6 boys each and 5 groups of 6 girls each) conducted in five junior high schools. The instrument was validated by five experts specialized in adolescent health and development, and pretested in a pilot study of 715 students.

### Bullying roles: participant roles in the bullying process

A description of bullying was provided to students to ensure consistency: “A student is being bullied when another student, or a group of students, say or do nasty and unpleasant things to him/her. It is also bullying when a student is teased or socially isolated in a way he or she doesn’t like. But it is not bulling when two students of about the same strength quarrel or fight.” [1, 6, 28]. The following single question was used to measure bullying experiences (bullying others or being bullied) both in school and away from school: “Have you ever encountered the following situations in the past year?” Response categories were “never”, “seldom: less than once a month”, “sometimes: more than once a month but less than once a week”, “often: once or twice a week” and “always: more than three times a week” (Appendix 1). In this study, we combined the three responses, sometimes, often and always, into one category which represented those who had had experiences of bullying [6, 28]. The other two responses were combined into one category, which represented no bullying experiences. Bullying experiences (in or outside of school) were then categorized into four groups according to bullying roles: (1) bullies, students whose bullying experiences involved them bullying others; (2) victims, students whose bullying experiences involved them being bullied; (3) bully/victims, students whose bullying experiences involved both bullying others and being bullied; (4) bystanders, students who were not directly involved in bullying experiences but were observers of bullying episodes as measured by the bystander experience scale. We defined a bystander as someone who was aware of, or saw an episode of bullying but was not either the bully or the victim [29–31].

### Defending behaviors

Students were asked to report how often they engaged in the following behaviors when they witnessed a bullying episode in the past year: reported it to the teacher, reported it to the disciplinary office of the school, stopped the perpetrator from hitting the victim, stopped the perpetrator from making fun of the victim, told others not to join in the bullying, comforted the victim, tried to help the victim, tried to be a peacemaker, and fetched people to come and help [8]. Response categories ranged from 1 (never) to 5 (always) (Appendix 2). The standardized Cronbach’s alpha for defending behaviors was 0.9. The score for defending behaviors was calculated by adding the scores of each item divided by the number of the items the students responded to. However, if a student responded to fewer than four items, then the student’s score was recorded as missing. A higher score indicated a higher frequency of defending behaviors.

### Mental health indicators

Two mental health indicators, social anxiety and depressive symptoms, were included in this study.

### Social anxiety score

There were seven items related to social anxiety in which the students were asked if they had had any of the following experiences in the past two weeks: feeling afraid to make new friends, worrying about being laughed at, feeling afraid to talk to strangers, feeling someone is laughing at you, feeling afraid someone is talking behind your back, feeling afraid others dislike you, and feeling afraid to perform or answer questions in public. This scale was developed based on the Social Anxiety Scale for Children (SASC-R) [32] and the Social Phobia and Anxiety Inventory for Children (SPAI-C) [33]. All the items were measured using a three-point scale with the possible responses of never (1), once or twice (2), or many times (3). The standardized Cronbach’s alpha was 0.82. We treated the score in the same way as the defending behaviors score.

### Depressive symptoms score

Regarding depressive symptoms, students rated how often in the past two weeks they had experienced the following emotions: didn’t feel like eating their favorite foods, felt very sad, cried for no reason, found it hard to carry out tasks, felt frightened, didn’t sleep well, lacked motivation, and felt gloomy and unhappy. This scale was designed based on the Center for Epidemiological Studies Depression Scale for Children (CES-DC) [34] and related literature [35, 36], and has been used in previous studies [37–39]. All items were measured using a three-point scale with the possible responses of never (1), once or twice (2), or many times (3). The standardized Cronbach’s alpha was 0.76. We treated the score in the same way as the defending behaviors score.

### Other variables

Based on past literature [1–3], factors associated with both bullying and mental health indicators were controlled for including student characteristics (sex, grade, aggressive tendency and communication skills) and family indicators (parents’ marital status, parents’ highest education level, family activity, family support, family conflict and level of punishment).

### Statistical analysis

Descriptive analysis was conducted to examine the distributions of the variables and followed by analysis of variance (ANOVA). Finally, a multivariate linear regression was performed to investigate the relationship between defending behaviors and mental health indicators whilst controlling for other variables. SAS 9.3 Survey Analysis procedures was used to conduct the above statistical analyses. Sampling weights were calculated from the inverse of the inclusion probabilities in consideration of sampling design effects.

Hypothesis 1 was tested using defending behaviors as the outcome variable and bullying roles as the predictor with adjustments made for several control variables. Hypothesis 2 was tested using mental health status as the outcome variable and defending behaviors as the predictor with or without bullying roles as a covariate along with other control variables. Hypothesis 3 was tested by stratifying the sample by bullying roles, then using mental health as the outcome variable and defending behaviors as the predictor, with adjustments made for other control variables.

## Results

### Distribution of bullying roles, mental health status, defending behaviors, and other related variables

Table 1 presents the distribution of bullying roles, mental health (depressive symptoms and social anxiety) status, defending behaviors and other related factors. In general, the majority of students were bystanders (80 %), 9.55 % were bullies, and 7.6 % were victims. Only 2.95 % were bully-victims. In general, participants rarely defended victims (mean score: 1.82) in the last year. The mean frequencies of social anxiety (mean score: 1.63) and depressive symptoms (means score: 1.65) were around once or twice in the previous week.

**Table 1:** Descriptions of main variables and other related variables (*N* = 2872)

	*N* (%)
[Main Variables]	
Bullying role: Bullies	256 (9.5)
Victims	218 (7.6)
Bully-victims	106 (2.9)
Bystanders	2,292 (80.0)
Defending behaviors score (range: 1–5)	1.82 (0.03)^a^
Social anxiety score (range: 1–3)	1.63 (0.01)^a^
Depressive symptoms score (range:1–3)	1.65 (0.01)^a^
[Other related Variables – student characteristics]	
Sex: Girl	1,430 (49.4)
Boy	1,442 (50.6)
Grade: 7	899 (31.7)
8	994 (33.4)
9	979 (34.9)
Aggressive tendency score (range: 1–5)	2.43 (0.03)^a^
Communication skills score (range: 1–4)	2.87 (0.02)^a^
[Other related Variables –parental characteristics]	
Parents’ marital status: Not married	567 (18.2)
Married	2,305 (81.8)
Parents’ education: Junior high school	450 (13.4)
Senior high school	1,664 (60.6)
College/University	500 (17.5)
Unknown	258 (8.5)
Family activities score (range:1–5)	2.89 (0.03)^a^
Family support score (range: 1–5)	3.12 (0.03)^a^
Family conflict score (range: 1–5)	1.73 (0.02)^a^
Punishment score (range: 1–5)	1.70 (0.02)^a^

*Note 1*: percentages (%) and means were weighted by sampling probabilities

*Note 2*: ^a^: Mean (S.D)

In terms of participant characteristics, the sample consisted of slightly more boys (50.6 %) than girls, with 31.7 % in the 7^th^ grade, 33.4 % in the 8^th^ grade, and 34.9 % in the 9^th^ grade. Participants had a moderate level of aggressive tendency and moderate communication skills.

In terms of parent characteristics, the majority of parents were married (81.8 %). Over half of parents had a senior high school level of education (60.3 %), 17.5 % had a college degree, 13.45 % had a junior high school level of education and 8.5 % had an unknown education level. Students perceived that the frequency of family activities was between seldom and sometimes, that the frequency of family support was between sometimes and often, and that the frequencies of family conflict and punishment were between never and seldom.

### Associations between defending behaviors and bullying roles

Table 2 shows the results of ANOVA and multivariate regression analysis for the associations between defending behaviors and bullying roles. The results of ANOVA showed that victims reported significantly higher defending behavior scores than bullies and bystanders. Multivariate analysis demonstrated that this finding persisted after controlling for other related variables.

**Table 2:** Associations between defending behaviors and bullying roles (*N* = 2,872)

	Defending behaviors score (Range: 1–5)					
	ANOVA		Multivariate regression			
	Mean	SD	Beta	STB	SD	*p*-value
Bullying role:						
Bullies (B)	1.82	0.06	−0.18	−0.06	0.07	0.011
Bystanders (BY)	1.80	0.03	−0.21	−0.10	0.06	0.002
Bully-victims (BV)	1.97	0.12	−0.07	−0.01	0.11	0.532
Victims (V)	2.02	0.07	ref			
	F = 3.24					
	(*p* =0.018)					
	Post-hoc test					
	V > B, BY					

*Note 1*: Means were weighted by sampling probabilities

*Note 2*: This multivariate regression model was adjusted for sex, grade, aggressive personality, communication skills, school attachment, parents’ marital status, parents’ education level, family activity level, family support level, family conflict level, and punishment level

### Associations between bullying roles, defending behaviors and mental health indicators

The association between bullying roles and social anxiety score is shown in Table 3. Using ANOVA and Post-hoc testing, social anxiety scores of victims and bully-victims were higher than bullies, followed by bystanders. After controlling for individual and family variables, victims and bully-victims still had higher social anxiety scores than that of bystanders. Defending behaviors were positively associated with social anxiety in the Pearson correlation test and multivariate regression modeling adjusting for bullying role and other variables.

**Table 3:** Associations between social anxiety, defending behaviors, and bullying roles (*N* = 2,872)

	Social anxiety score (Range: 1–3)										
	Bivariate analysis			Multivariate regression I				Multivariate regression II			
	Mean	SD	Test	Beta	STB	SD	*p*-value	Beta	STB	SD	*p*-value
Bullying role:											
Bullies (B)	1.68	0.04	F = 20.80	0.01	0.00	0.23	0.98	−0.00	−0.00	0.23	0.9898
Victims (V)	1.89	0.04	(*P* = 0.018)	1.73	0.13	0.26	<.0001	1.67	0.13	0.26	<.0001
Bully-victims (BV)	1.90	0.07	Post-hoc test	1.58	0.08	0.46	<.0001	1.54	0.08	0.45	0.0009
Bystanders (BY)	1.59	0.01	V, BV > B > B	ref				ref			
Defending behaviors score	*r* = 0.053		*P* = 0.0041	-				0.27	0.07	0.13	0.02

*Note 1*: Means were weighted by sampling probabilities

*Note 2*: The two multivariate regression models were adjusted for sex, grade, aggressive personality, communication skills, school attachment, parents’ marital status, parents’ education level, family activity level, family support level, family conflict level, and punishment level. Model I examined the relationship between bullying roles and social anxiety. Model II examined the relationship between bullying roles and social anxiety after adjusting for defending behaviors

The association between bullying role and depressive symptoms score is shown in Table 4. Using ANOVA and Post-hoc testing, the depressive symptoms score of bullies, victims and bully-victims were all higher than that of bystanders. After controlling for individual and family variables, only victims had a higher depressive symptoms score than that of bystanders. Defending behaviors were positively associated with depressive symptoms score in the Pearson correlation test and multivariate regression modeling adjusting for bullying role and other variables.

**Table 4:** Associations between depressive symptoms, defending behaviors, and bullying roles (*N* = 2,872)

	Depressive symptoms score (Range: 1–3)										
	Bivariate analysis			Multivariate regression I				Multivariate regression II			
	Mean	SD	Test	Beta	STB	SD	*p*-value	Beta	STB	SD	*p*-value
Bullying experience:											
Bullies (B)	1.80	0.03	F = 22.37	0.47	0.05	0.25	0.06	0.46	0.04	0.25	0.067
Victims (V)	1.85	0.04	*p* < .0001	1.28	0.11	0.24	<.0001	1.22	0.11	0.24	<.0001
Bully-victims (BV)	1.78	0.04	Post-hoc test	0.38	0.02	0.24	0.11	0.34	0.02	0.24	0.152
Bystanders (BY)	1.61	0.01	B, V, BV > BY	ref				ref			
Defending behavior score	*r* = 0.047		*P* = 0.0115	-				0.27	0.07	0.10	0.008

*Note 1*: Means were weighted by sampling probabilities

*Note 2*: This model was adjusted for sex, grade, aggressive personality, communication skills, school attachment, parents’ marital status, parents’ education level, family activity level, family support level, family conflict level, and punishment level. Model I examined the relationship between bullying roles and depressive symptoms. Model II examined the relationship between bullying roles and depressive symptoms after adjusting for defending behaviors

### Associations between defending behaviors and mental health indicators stratified by bullying roles

Table 5 presents the relationship between defending behaviors and mental health indicators stratified by bullying roles. Among bullies and bully-victims, defending behavior scores were not associated with mental health indicators after adjustment for other related variables. However in victims, higher defending behaviors was associated with a higher depressive symptom score. In bystanders, higher defending behaviors was associated with higher scores for both depressive symptoms and social anxiety.

**Table 5:** The associations between defending behaviors and mental health indicators stratified by bullying roles

Social anxiety score																
	1. Bullies (*n* = 256)				2. Victims (*n* = 218)				3. Bully-victims (*n* = 106)				4. Bystanders (*n* = 2292)			
	Beta	STB	S.D.	*P*-value	Beta	STB	S.D.	*P*-value	Beta	STB	S.D.	*P*-value	Beta	STB	S.D.	*P*-value
Defending behaviors	−0.02	−0.04	0.05	0.654	0.05	0.08	0.08	0.554	0.02	0.03	0.11	0.888	0.05	0.09	0.02	0.005
Depressive symptoms score																
	1. Bullies (*n* = 256)				2. Victims (*n* = 218)				3. Bully-victims (*n* = 106)				4. Bystanders (*n* = 2292)			
	Beta	STB	S.D.	*P*-value	Beta	STB	S.D.	*P*-value	Beta	STB	S.D.	*P*-value	Beta	STB	S.D.	*P*-value
Defending behaviors	0.02	0.04	0.04	0.578	0.12	0.23	0.04	0.011	0.02	0.04	0.09	0.838	0.04	0.07	0.01	0.005

*Note 1*: *: *p* < .05, **: *p* < .01, ***: *p* < .001

*Note 2*: All models were adjusted for sex, grade, aggressive personality, communication skills, school attachment, parents’ marital status, parents’ education level, family activity level, family support level, family conflict level, and punishment level

## Discussion

Defending behaviors were associated with bullying roles, with victims reporting higher defending behavior scores than bullies or bystanders. Victims and bully-victims also had greater social anxiety than bullies or bystanders. Bystanders had lower depressive symptoms than bullies, victims and bully-victims. Defending behaviors were positively associated with social anxiety and depressive symptoms after adjusting for bullying role and other related factors. After stratifying by bullying role, defending behaviors were positively associated with social anxiety in bystanders, and were positively associated with depressive symptoms in victims and bystanders.

### Victims reported more defending behaviors than bystanders and bullies

All four bullying roles (bullies, victims, bully-victims, and bystanders) reported some level of defending behaviors. In other words, it is not only bystanders, but also other involvers that may defend victims. However, victims reported more defending behaviors than bystanders and bullies after controlling for other variables. There are several possible explanations for this finding. First, victims probably have a higher level of empathy and anti-bullying sentiment compared to bullies and bystanders. Thornberg [40] found that students who feel empathy for victims or students who believe that bullying is morally wrong are more motivated to intervene in bullying situations. The past victimization experiences of victims may make some victims feel more empathy for other victims and their experiences may also make them believe firmly that bullying is wrong. However, we have no data on the empathy or bullying beliefs associated with particular bullying roles so are unable to explore this further. Second, victims are probably more sensitive to the signs of bullying than bullies and bystanders. According to the process of intervention proposed by Latané and Darley [41], the witness first has to be aware of the situation, interpret the situation as dangerous, consider self-responsibility, decide what to do, and finally implement an action. The victimization experiences of victims make them more familiar with the signs of bullying, and they may be better able to notice bullying than bullies or bystanders, and hence more likely to intervene. However, we have no data in our study on differences in the ability to notice bullying between victims, bystanders or bullies. Further research is needed to better understand the internal struggles and decision-making processes involved in the defending behaviors of victims.

### Positive associations between defending behaviors, social anxiety and depressive symptoms

We found that defending behaviors were positively associated with social anxiety and depressive symptoms. This is probably the result of pro-bullying norms in the classroom. The power imbalance between bullies and victims can result in witnesses also taking the side of bullies as they may find it easier to side with the more powerful social group member [7, 42]. Under such pro-bullying norms, if witnesses intervene to help victims he/she may end up being socially isolated. As social isolation has a known association with mental health outcomes [43], this increased risk of social isolation from defending behavior could explain the association between defending behavior and worse mental health.

### The association between defending behaviors and mental health indicators varied by bullying roles

Based on the results of the stratified analysis, we clarified the associations between defending behaviors and mental health indicators for the four bullying roles. In this study, the majority of students (80 %) were bystanders whose defending behavior score was positively associated with social anxiety and depressive symptoms. Previous studies have noted that an uncomfortable tension (cognitive dissonance) can arise from observing others being bullied and not intervening [16, 42]. However, in this study, we found that both not intervening and intervening were associated with emotional disturbance.

Defending behaviors in bullies were not associated with social anxiety or depressive symptoms. Bullies often possess a higher social status than victims or bystanders [7, 16, 42] and high social status is necessary for defending victims [7]. Bullies and defenders have some characteristics in common such as being popular and having high self-esteem. Therefore, bullies who intervened to help victims may not experience peer pressure to the same extent as others and therefore may also not experience negative mental health effects.

Defending behaviors in victims were positively associated with depressive symptoms, but not social anxiety. Victims may experience the greatest negative health impact if they are the only target in the class [7]. If victims help other victims, this action would allow them to ally themselves with other victims. In other words, they could make friends by helping others. Therefore, defending other victims may not increase social anxiety in victims but may lead to increased depressive symptoms. This could be because the victim who defends others still has to confront pro-bullying peer pressure even if they are not the only victim in the class.

Similar to bullies, defending behaviors were not associated with social anxiety or depressive symptoms in bully-victims. However, we think the mechanism through which defending behaviors affect mental health in bully-victims may not be the same as that in bullies. Bully-victims report more conduct disorders and interpersonal relationship problems than pure bullies and pure victims. They lack the power of bullies and are vulnerable like victims. Olweus (1993) [1] classified bully-victims as counter-strike victims due to their lack of social skills and power.

### Study limitations

This study is a cross-sectional study, which limits the interpretation of causal effects. In addition, some important potential confounders were not controlled for including peer attitudes toward bullying, perceived peer pressure and empathy toward victims. Therefore, the mechanisms through which defending behaviors impact on mental health will need to be confirmed by further studies. The particular form of bullying (such as physical bullying, verbal bullying, relational bullying or cyberbullying) and whether the associations between defending behaviors and mental health indicators vary according to the form of bullying were not discussed in this study and should be 7investigated in the future. In addition, the assessment of bullying would have been more comprehensive if additional measures such as reports from peers, teachers or parents were provided. Although in this study bullying measures were based on self-report which can lead to underreporting, self-reported measures of bullying have been found to have acceptable reliability and validity [44].

## Conclusion

Despite its limitations, the present study is the first study to investigate the association between defending behaviors and mental health status by different bullying roles. We found that victims had a higher frequency of defending behaviors than bystanders. Higher defending behaviors was associated with higher social anxiety and depressive symptoms. In bystanders, defending behaviors were positively associated with social anxiety and depressive symptoms. In bullies, no significant association between defending behaviors and mental health indicators was observed.

The important contribution of this study is the provision of empirical data on the relationship between defending behaviors and mental health, which can be used to inform the development of future intervention strategies. Based on our results, we recommend the development of strategies to minimize social anxiety and depression in victims and bystanders when developing anti-bullying interventions. Since bullies experience less stress than the other players, it might also be feasible to incorporate strategies that encourage students with a bullying tendency to intervene and defend their more vulnerable peers. This could lead to increased defending behaviors and the reduction of bullying in schools. Future studies should involve long-term follow-up to verify the temporal sequence and causal effects. Some defending behaviors may be more effective than others in terms of their impact on bullying. It is possible that the association between defending behavior and mental health varies according to the effectiveness of defending behavior and this should be explored in future research. The mechanism through which defending behaviors impact on mental health should also be explored further.

## Figures and Tables

**Table 6:**
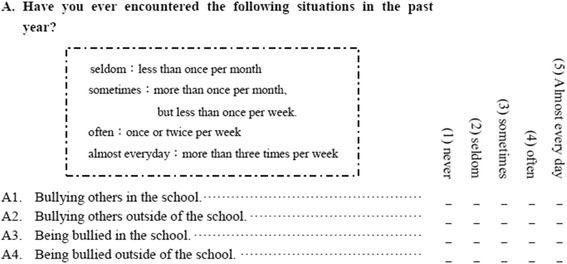
Questions about bullying experiences

## Appendix 2

**Table 7:** Questions about defending behaviors

B. When you saw someone being bullied, how did you react?		(1) never	(2) seldom	(3) sometimes	(4) often	(5) Almost every day
B1.	When someone has been bullied, I will tell the teacher.	□	□	□	□	□
B2.	When someone has been bullied, I will tell the school.	□	□	□	□	□
B3.	When someone hits somebody, I will stop them.	□	□	□	□	□
B4.	When someone makes fun of somebody, I will stop them.	□	□	□	□	□
B5.	I will tell my classmates not to join in with bullying.	□	□	□	□	□
B6.	I will comfort victims of bullying.	□	□	□	□	□
B7.	I will try to help victims of bullying.	□	□	□	□	□
B8.	I will be a peacemaker and try to mediate in difficult situations.	□	□	□	□	□
B9.	I will find someone to help bullying victims.	□	□	□	□	□
